# Recovery of children following hospitalisation for complicated severe acute malnutrition

**DOI:** 10.1111/mcn.13302

**Published:** 2021-12-22

**Authors:** Mutsa Bwakura‐Dangarembizi, Cherlynn Dumbura, Beatrice Amadi, Bernard Chasekwa, Deophine Ngosa, Florence D. Majo, Jonathan P. Sturgeon, Kanta Chandwe, Chanda Kapoma, Claire D. Bourke, Ruairi C. Robertson, Kusum J. Nathoo, Robert Ntozini, Shane A. Norris, Paul Kelly, Andrew J. Prendergast

**Affiliations:** ^1^ University of Zimbabwe College of Health Sciences Harare Zimbabwe; ^2^ Zvitambo Institute for Maternal and Child Health Research Harare Zimbabwe; ^3^ University of Witwatersrand Johannesburg South Africa; ^4^ Tropical Gastroenterology and Nutrition Group, University of Zambia Lusaka Zambia; ^5^ Blizard Institute, Queen Mary University of London London UK

**Keywords:** HIV, morbidity, nutritional recovery, readmission, severe acute malnutrition

## Abstract

Nutritional recovery and hospital readmission following inpatient management of complicated severe acute malnutrition (SAM) are poorly characterised. We aimed to ascertain patterns and factors associated with hospital readmission, nutritional recovery and morbidity, in children discharged from hospital following management of complicated SAM in Zambia and Zimbabwe over 52‐weeks posthospitalization. Multivariable Fine‐Gray subdistribution hazard models, with death and loss to follow‐up as competing risks, were used to identify factors associated with hospital readmission; negative binomial regression to assess time to hospitalisation and ordinal logistic regression to model factors associated with nutritional recovery. A total of 649 children (53% male, median age 18.2 months) were discharged to continue community nutritional rehabilitation. All‐cause hospital readmission was 15.4% (95% CI 12.7, 18.6) over 52 weeks. Independent risk factors for time to readmission were cerebral palsy (adjusted subhazard ratio (aSHR): 2.96, 95% CI 1.56, 5.61) and nonoedematous SAM (aSHR: 1.64, 95%CI 1.03, 2.64). Unit increases in height‐for‐age Z‐score (HAZ) (aSHR: 0.82, 95% CI 0.71, 0.95) and enrolment in Zambia (aSHR: 0.52, 95% CI 0.28, 0.97) were associated with reduced subhazard of time to readmission. Young age, SAM at discharge, nonoedematous SAM and cerebral palsy were associated with poor nutritional recovery throughout follow‐up. Collectively, nonoedematous SAM, ongoing SAM at discharge, cerebral palsy and low HAZ are independent risk factors for readmission and poor nutritional recovery following complicated SAM. Children with these high‐risk features should be prioritised for additional convalescent care to improve long‐term outcomes.

## Key messages

- One‐in‐six children managed for SAM were readmitted into hospital over the first year after discharge and one‐in‐eight remained undernourished by 52 weeks of follow‐up.
- Nonoedematous SAM, ongoing SAM at the time of discharge and underlying cerebral palsy were independent risk factors for hospital readmission and poor nutritional recovery.
- Low HAZ was a risk factor for hospital readmission and poor nutritional recovery.
- Postdischarge care should focus on children with disability, nonoedematous SAM at initial hospitalisation and have ongoing SAM at the time of discharge.
- Stunting should be considered in the management of children with SAM.

## Graphical abstract

Nutritional recovery and hospital readmission following inpatient management of complicated severe acute malnutrition (SAM) are poorly characterised. We aimed to ascertain patterns and factors associated with hospital readmission, nutritional recovery and morbidity, in children discharged from hospital following management of complicated SAM in Zambia and Zimbabwe over 52‐weeks posthospitalization. Nonoedematous SAM, ongoing SAM at discharge, cerebral palsy and low height‐for‐age Z‐score were found to be independent risk factors for readmission and poor recovery following complicated SAM.

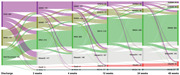

## 1 INTRODUCTION

Severe acute malnutrition (SAM) is defined by the presence of bilateral nutritional oedema, severe wasting (weight for height Z‐score (WHZ) <−3), and/or mid‐upper arm circumference (MUAC) <115 mm, and is associated with a high risk of death, mainly from infections (Rytter et al., 2014). An estimated 16.4 million children below 5 years of age were affected by SAM globally in 2017 (World Bank, World Health Organisation & United Nations Children's Fund, 2018). The vicious cycle of malnutrition and infection is well recognised, and often leads to clinical complications (Katona & Katona‐Apte, 2008; Rytter et al., 2014). Complicated SAM, which is characterised by clinical instability and lack of appetite, is managed in inpatient facilities where medical complications and acute infections are treated (World Health Organisation, 2013). The goals of managing SAM are to prevent short‐term morbidity and mortality, achieve sustained nutritional recovery, and support long‐term neurocognitive development (Bhutta et al., 2017). Discharge from hospital to continue outpatient therapeutic feeding is based on return of appetite and resolution of medical complications rather than improvements in anthropometry. Subsequent discharge from outpatient treatment programmes occurs when there is adequate nutritional recovery (defined as WHZ > −2, or MUAC > 125 mm and/or resolution of oedema for 2 weeks) (World Health Organisation, 2013).

Following discharge from in‐patient treatment for complicated SAM, children have persistent vulnerabilities, leading to an ongoing high mortality risk (Lenters et al., 2013). Contributing factors include immune impairment, chronic infections such as tuberculosis (TB) and HIV, and a return to the same poor social environment (Ngari et al., 2018). Data from the Health Outcomes, Pathogenesis and Epidemiology of Severe Acute Malnutrition (HOPE‐SAM) cohort in Zimbabwe and Zambia showed that 43.9% of children had ongoing SAM (by WHO criteria) at the time of discharge from hospital and these children had more than twofold increased risk of dying in the first year postdischarge, compared to children who had anthropometric recovery at discharge. Children with HIV infection had threefold higher mortality than children with SAM alone, and this mortality risk persisted despite antiretroviral therapy (ART). Nonoedematous SAM and cerebral palsy were additional risk factors for postdischarge mortality in this cohort (Bwakura‐Dangarembizi et al., 2021).

Achieving good short‐term and long‐term health outcomes after treatment of SAM is challenging, especially in poorly resourced settings with fragile health systems (Khanum et al., 1998; Stobaugh, Rogers, et al., 2018; Wiens et al., 2013). Few studies have systematically mapped patterns of nutritional recovery and morbidity after discharge from hospital following management of complicated SAM. Understanding the factors associated with nutritional recovery and hospital readmission would help identify the most vulnerable children at the time of discharge and may inform additional interventions to improve outcomes. Here, we present further data from the HOPE‐SAM cohort, in which children were followed for 1 year after hospital discharge following management of complicated SAM. Our hypotheses were, first, that HIV‐positive compared to HIV‐negative children with SAM have earlier and more frequent hospital readmissions, more illness episodes and poorer nutritional recovery, despite the availability of ART and, second, that a combination of biological and social risk factors predict readmission and nutritional recovery.

## 2 METHODS

### 2.1 Study design

HOPE‐SAM was an observational cohort study that enroled children under 5 years of age hospitalised for complicated SAM at three tertiary referral hospitals in Lusaka, Zambia and Harare, Zimbabwe between August 2016 and March 2018 (Bwakura‐Dangarembizi et al., 2019). The protocol, standard operating procedures and case report forms (CRFs) are available at https://osf.io/29uaw/. Here, we report readmissions, morbidity and nutritional recovery over the first‐year postdischarge and factors associated with these outcomes.

### 2.2 Study population

Eligible children were aged below 60 months, admitted at three tertiary hospitals in the two countries with SAM, defined as WHZ <−3, MUAC < 115 mm and/or the presence of nutritional oedema in children above 6 months of age (and WHZ <−3 or nutritional oedema in those below 6 months) (World Health Organisation, 2013). Primary caregivers gave written informed consent for their children to participate in the study.

### 2.3 Study procedures and follow‐up

Study procedures have been previously described (Bwakura‐Dangarembizi et al., 2019). We used standardised CRFs to collect baseline clinical, demographic, household and caregiver data. Child HIV status was determined using rapid antibody or DNA polymerase chain reaction (PCR) testing according to age. Inpatient care was provided by the ward clinical teams according to WHO‐adapted country‐specific guidelines for the management of complicated SAM. A study physician reviewed participants daily during hospitalisation to document progress. Discharge was based on clinical recovery (World Health Organisation, 1999, 2013).

Postdischarge care was provided outside of the study and followed WHO‐adapted country‐specific guidelines. Caregivers were given a supply of ready‐to‐use therapeutic food (RUTF) (usually 2 weeks, depending on availability) and counselled on how to feed the child. RUTF was dispensed until children achieved moderate acute malnutrition (MAM) status on anthropometry; however, in Zimbabwe, there were frequent stock‐outs of RUTF in the urban and peri‐urban areas. In Zimbabwe, once children attained MAM status, RUTF was stopped and no supplementary food was available; caregivers were counselled on using locally available food. In Zambia, children with MAM received High Energy Protein Supplement (HEPS) or Corn Soya Blend and the caregiver was counselled on how to prepare HEPS porridge and the appropriate frequency of feeds, in addition to nutritional counselling on ‘what to give, how much and how often’ according to the Supplementary Feeding Programme for moderately malnourished children.

Children were seen in a dedicated study clinic at 2, 4, 12, 24 and 48 weeks postdischarge, with window periods around each time‐point. Endline visits, therefore, occurred at a median of 49.3 (48.1, 51.4) weeks postdischarge, with 21.3% of attending children seen by 48 weeks and 84.2% of children seen by 52 weeks. Endline visits for all analyses were censored at 52 weeks.

Anthropometric measurements were undertaken using standardised methods at study enrolment, hospital discharge and at each of five scheduled follow‐up visits. Nutritional status was classified as adequately nourished (WHZ ≥ −2 and/or MUAC ≥ 125 mm), MAM (WHZ between ‐2 and −3 and/or MUAC of 115–124 mm) and SAM (WHZ <−3 or MUAC < 115 mm, or bilateral nutritional oedema) (World Health Organisation, G. 2013). If WHZ score and MUAC classified children differently, the lower nutritional category was used. Morbidity was assessed by all‐cause readmission into hospital and caregiver‐reported symptoms in the previous 2 weeks at each study visit. Details of the clinic and hospital visits were verified during study visits from caregivers' hand‐held child health cards. RUTF and ART were provided from government clinics and admitting hospitals. Referrals were made from the study clinic for hospital readmission when indicated. When a child failed to attend the study clinic for their scheduled visit, phone calls were made to reschedule the visit; children missing multiple study visits were visited at home where possible.

### 2.4 Sample size

We aimed to enrol a minimum of 600 and a maximum of 800 children with complicated SAM. We estimated that mortality of 15% and overall loss to follow‐up of 15% would provide 560 evaluable children at 1 year, of whom 224 would have HIV‐SAM based on an estimated prevalence of 40% in hospitalised children. This would provide >80% power to detect absolute differences of 17% in binary outcomes between HIV‐positive children and HIV‐negative children with SAM, and of 0.33 standard deviations in continuous outcomes.

### 2.5 Statistical methods

A pre‐specified analysis plan is available at https://osf.io/29uaw/. Data collected onto paper CRFs were double‐entered onto the study database. Anthropometric Z‐scores were calculated using 2006 WHO growth standards. Baseline variables were compared between HIV‐positive and HIV‐negative children using *χ* ^2^ or Fisher's exact tests for categorical variables, and *t* tests or Mann–Whitney tests for continuous variables, depending on distributions. Morbidity, readmission and nutritional recovery analyses were conducted 52 weeks postdischarge. Analyses were conducted using STATA version 14.0 (StataCorp.).

#### 2.5.1 Time to first hospital readmission

Fine‐Gray subdistribution hazard models were used to estimate the hazard ratios for hospital readmission, with death and loss to follow‐up as competing risks. We also ran a Cox proportional hazards model, assuming no competing risks, as a sensitivity analysis. Cumulative incidence curves were used to describe the time to first hospital readmission. The selection of exposure variables was based on biological plausibility. The full list of variables is provided in Supporting Informations. Strength of association with time to first hospital readmission was initially assessed using univariable analyses. Variables significant at *p* < 0.25 were included in a multivariable competing risks regression model together with a set of pre‐specified variables that were forced to remain in the model: age, sex, country, oedema on admission, SAM at discharge, cerebral palsy and HIV status. Variables with *p* < 0.05 in the final model were deemed associated factors.

#### 2.5.2 The incident rate of hospital readmissions

Negative binomial regression models which account for overdispersion were used to estimate the incidence rate of readmissions. Risk factors for readmission were identified using the same approach as for the time to first hospital readmission.

#### 2.5.3 Morbidity analysis

The following morbidity outcomes were assessed at each study visit by caregiver recall: fever, diarrhoea, respiratory symptoms (defined as cough or difficult breathing), poor appetite and weight loss. The period prevalence of illness episodes was estimated as the proportion of children with any caregiver‐reported symptom in the 2 weeks preceding each visit, stratified by HIV status.

#### 2.5.4 Nutritional recovery

Nutritional status was assessed at five‐time points postdischarge using regression models with WHZ and MUAC as outcomes. Ordinal logistic regression was also used to identify factors associated with nutritional recovery, defined as moving from a lower to a higher nutritional category (i.e., from SAM to either MAM or adequately nourished and from MAM to adequately nourished). Exposure variables were chosen in the same way as those for hospitalisation.

### 2.6 Ethical considerations

Ethical approval was obtained from the University of Zambia Biomedical Research Ethics Committee, and the Medical Research Council of Zimbabwe. The ethics committee of the Queen Mary University of London provided an advisory review.

## 3 RESULTS

### 3.1 Study enrolment

A total of 755 children with SAM were enroled between 13 July 2016 and 31 March 2018; Figure S1 summarises their enrolment and follow‐up. Five children were excluded due to co‐enrolment in other studies or ineligibility. Among 750 eligible children, two died and three withdrew consent before baseline data collection, leaving 745 children in the study. Seventy children died during their initial hospitalisation and 26 exited the study before discharge, leaving 649 children who were discharged from the hospital and included in these analyses. Fifty‐five children died and 21 withdrew consent during postdischarge follow‐up. Postdischarge follow‐up at each scheduled study visit ranged between 72.8% and 78.2% (Figure S1).

### 3.2 Discharge characteristics

Child, caregiver and household characteristics are shown in Table 1. The median age of children at discharge from the hospital was 18.2 months (IQR: 13.6, 22.6), with 53% males; 20% of children were HIV‐positive. Sixty‐five percent of children had oedematous malnutrition on admission to hospital and 78% were stunted (HAZ <−2). HIV‐positive compared to HIV‐negative children were significantly older, more wasted, underweight and stunted and more likely to have had previous SAM or persistent diarrhoea; they were also hospitalised for longer, were more anaemic and were more likely to be discharged on TB medication (Table 1). Only 25% of the cohort were breastfeeding at the time of hospital discharge; 31.4% had stopped before 12 months of age (at median 8 months; IQR: 6, 9). Nearly all (93.2%) children were cared for by their biological mother, who had median of 11 years of education (IQR: 10, 11). Seventy‐one (10.9%) children discharged from hospital never attended a subsequent follow‐up visit; these children had significantly younger mothers than those attending at least one follow‐up visit (median 23 vs. 27 years, *p* < 0.001) and were less likely to be on TB medication (5.6% vs. 14.5%, *p* = 0.04) but were otherwise similar to the follow‐up cohort (Table S1).

**Table 1:** Child, caregiver and household characteristics at hospital discharge

	All (*N* = 649)	HIV‐positive (*N* = 130)	HIV‐negative (*N* = 519)	*p* value^a^
Country				
• Zambia	188/649 (29.0%)	56/130 (43.1%)	132/519 (25.4%)	<0.001
□ Zimbabwe	461/649 (71.0%)	74/130 (56.9%)	387/519 (74.6%)	
Age, months; median (IQR)	18.2 (13.6, 22.6)	20.0. (14.5, 24.9)	17.5 (13.4, 22.1)	<0.001
Male	344/649 (53.0%)	66/130 (50.8%)	278/519 (53.6%)	0.57
Oedematous SAM at hospitalisation	422/649 (65.0%)	67/130 (51.5%)	355/519 (68.4%)	<0.001
Stunted (HAZ <−2)	504/649 (77.7%)	117/130 (90.0%)	387/519 (74.6%)	<0.001
Anthropometry				
□ WHZ, mean (SD)	−2.2 (1.5)	−2.4 (1.4)	−2.1 (1.5)	0.05
□ WAZ, mean (SD)	−3.3 (1.6)	−3.7 (1.2)	−3.2 (1.7)	0.002
□ HAZ, mean (SD)	−3.1 (1.5)	−3.5 (1.2)	−3.0 (1.6)	0.003
□ MUAC, cm; mean (SD)	12.3 (1.6)	11.7 (1.4)	12.4 (1.6)	<0.001
Past history				
• Complicated SAM	92/638 (14.4%)	30/129 (23.3%)	62/509 (12.2%)	0.001
□ Uncomplicated SAM	124/622 (19.9%)	40/124 (32.3%)	84/498 (16.9%)	<0.001
□ Persistent diarrhoea (past 14 days)	352/649 (54.2%)	84/130 (64.6%)	268/519 (51.6%)	0.008
Breastfeeding				
□ Currently breastfeeding	165/649 (25.4%)	30/130 (23.1%)	135/519 (26.0%)	0.49
□ Premature breastfeeding cessation (<12months of age)	162/516 (31.4%)	29/105 (27.6%)	133/411 (32.4%)	0.35
□ Duration of breastfeeding, months; median (IQR)	8 (6, 9)	8 (6, 9)	8 (6, 9)	0.90
Duration of hospitalisation, days; median (IQR)	7 (4, 12)	10 (6, 17)	7 (4, 11)	<0.001
Haemoglobin, g/dl; median (IQR)^b^	9.3 (8.2, 10.3)	8.8 (7.7, 9.6)	9.4 (8.3, 10.4)	<0.001
Chronic underlying conditions				
□ Cerebral palsy	30/649 (4.6%)	1/130 (0.8%)	29/519 (5.6%)	0.02
□ Hydrocephalus	3/649 (0.5%)	0/130 (0%)	3/519 (0.6%)	0.39
□ Congenital heart disease	14/649 (2.2%)	1/130 (0.8%)	13/519 (2.5%)	0.22
Medications at discharge				
□ TB medication	88/649 (13.6%)	40/130 (30.8%)	48/519 (9.2%)	<0.001
□ ART medication	N/A	66/130 (50.8%)	N/A	N/A
Primary caregiver characteristics				
Relationship to the child				
□ Mother	590/633 (93.2%)	116/125 (92.8%)	474/508 (93.3%)	0.84
□ Mother's age, years; median (IQR)	26 (22, 30)	28 (23, 32)	26 (22, 31)	0.19
Caregiver marital status				
□ Married/stable union	471/633 (74.4%)	88/125 (70.4%)	383/508 (75.4%)	0.25
Caregiver education, years; median (IQR) caregiver employment				
□ None	373/630 (59.2%)	67/125 (53.6%)	306/505 (60.6%)	0.07
□ Skilled	46/630 (7.3%)	6/125 (4.8%)	40/505 (7.9%)	
□ Unskilled	211/630 (33.5%)	52/125 (41.6%)	159/505 (31.5%)	
Household characteristics				
Place of residence				
□ Rural	99/645 (15.3%)	20/130 (15.4%)	79/515 (15.3%)	0.02
□ Urban	404/645 (62.6%)	70/130 (53.8%)	102/515 (19.8%)	
□ Peri‐urban	142/645 (22.0%)	40/130 (30.7%)	334/515 (64.9%)	
Household drinking water source				
□ Improved	599/641 (93.4%)	123/130 (94.6%)	476/511 (93.2%)	0.54
Household toilet facilities				
□ Improved	568/643 (88.3%)	105/129 (81.4%)	463/514 (90.1%)	0.01
□ Unimproved	51/643 (7.9%)	18/129 (14.0%)	33/514 (6.4%)	
□ None	24/643 (3.7%)	6/129 (4.7%)	18/514 (3.5%)	
Household electricity				
Yes	295/634 (46.5%)	55/126 (43.7%)	240/508 (47.2%)	0.47

*Note*: Data are n/N (column %) unless otherwise stated.

Abbreviations: ART, antiretroviral therapy; HAZ, height‐for‐age Z‐score; IQR, Interquartile range; MUAC, mid‐upper arm circumference; N/A, not applicable; SAM, severe acute malnutrition; SD, standard deviation; TB, tuberculosis; WAZ, weight‐for‐age Z score; WHZ, weight‐for‐height Z score.

^a^ *p* value comparing HIV‐positive and HIV‐negative groups at discharge.

^b^ Haemoglobin was measured at discharge; if no discharge haemoglobin measurement was available, the value measured at the time point closest to discharge was used.

### 3.3 Rates of hospital readmission

Table S2 shows the unadjusted and adjusted incident rate ratios for hospital readmission for each explanatory variable. Among the 89 children who were readmitted to the hospital, 25 (28.1%) were admitted more than once and 13/89 (14.6%) died. We found two factors to be independently associated with readmission incidence: nonoedematous SAM and HAZ. Children with nonoedematous SAM had a 65% increased readmission rate compared to those with oedematous SAM (aIRR: 1.65, 95% CI: 1.05, 2.61). Better linear growth was associated with a lower rate of readmission: for every unit increase in HAZ there was a 19% lower rate of hospital readmission (aIRR: 0.81, 95% CI: 0.71, 0.94). There was some evidence of lower readmission rate for children in Zambia versus Zimbabwe (aIRR: 0.62, 95% CI: 0.37, 1.05) and higher readmission rate among children with cerebral palsy (aIRR: 2.02, 95% CI: 0.89, 4.59). No other factors predicted hospital readmission rate.

### 3.4 Time to first hospitalisation

**Figure 1:**
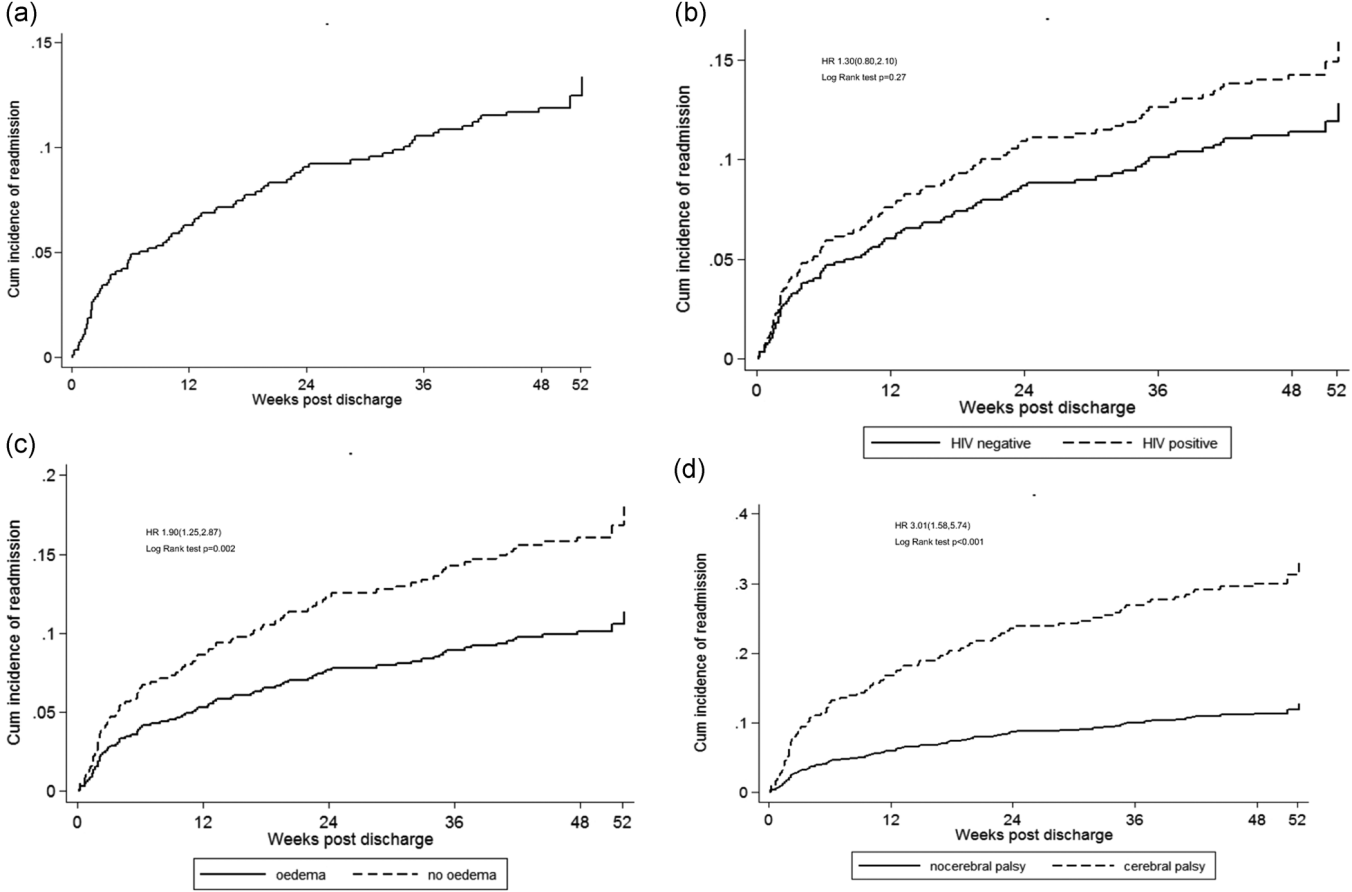
Cumulative incidence readmission among children discharged from hospital after treatment for complicated SAM. Curves showing cumulative incidence of readmission (death and loss to follow up as competing risks) postdischarge and log rank test *p* value. (a) overall cumulative incidence of readmission; (b) cumulative incidence of readmission by child HIV status; (c) cumulative incidence of readmission among children with oedematous versus nonoedematous SAM at the time of initial hospitalisation; (d) cumulative incidence of readmission in children with cerebral palsy versus those without cerebral palsy. Univariable subhazard ratios and log rank test *p* values were used to compare the differences in time to readmission between groups. Adjusted subhazard ratios are shown in Table 2

Of the 649 children discharged from the hospital, data on readmission were available for 578 (89%). Of these, 89 (15.4%; 95% CI: 12.7, 18.6) were readmitted through Week 52, with 52.8% of admissions occurring in the first 12 weeks after discharge (Figure 1a). Time to readmission did not differ significantly between HIV‐positive and HIV‐negative children (Figure 1b). We found four factors that were independently associated with time to first hospital readmission, using both the Fine‐Gray hazard model which accounted for death and loss to follow up as competing risks and the Cox proportional hazards model (Table 2). Nonoedematous compared to oedematous SAM was associated with a 64% increased subhazard of readmission (aSHR: 1.64, 95% CI: 1.03, 2.64; Figure 1c). Unit increases in HAZ were associated with an 18% lower subhazard of readmission (aSHR: 0.82, 95% CI: 0.71, 0.95); this association remained even after adjusting for WHZ (data not shown). Children with cerebral palsy were three times more likely to be readmitted (aSHR: 2.96, 95% CI: 1.56, 5.61; Figure 1d). Finally, there was evidence that children enroled in Zambia had a longer time period to hospital readmission compared to those enroled in Zimbabwe (aSHR: 0.52, 95% CI: 0.28, 0.97).

**Table 2:** Factors associated with time to readmission

	Never hospitalised (*N* = 560 (%))	Hospitalised (*N* = 89(%))	Cox proportional hazards model for time to readmission	*p* value	Competing risk regression model time to readmission aSHR (95% CI)	*p* value
Country						
□ Zimbabwe	392/461 (85.0%)	69/461 (15.0%)	Reference	0.06	Reference	0.04
□ Zambia	168/188 (89.4%)	20/188 (10.6%)	0.60 (0.35, 1.01)		0.52 (0.28,0.97)	
Sex						
□ Male	289/344 (84.0%)	55/344 (16.0%)	Reference	0.20	Reference	0.13
□ Female	271/305 (88.9%)	34/305 (11.1%)	0.75 (0.48, 1.17)		0.70 (0.45,1.11)	
Age at discharge (months), median (IQR)	18.4 (13.8, 22.5)	17.2 (11.8, 23.4)	0.98 (0.96, 1.01)	0.23	0.99 (0.95,1.02)	0.36
HIV status						
□ Negative	452/519 (87.1%)	67/519 (12.9%)	Reference	0.24	Reference	0.41
□ Positive	108/130 (83.1%)	22/130 (16.9%)	1.37 (0.81, 2.32)		1.27 (0.72,2.25)	
Type of SAM at hospitalisation						
□ Oedematous	377/422 (89.3%)	45/422 (10.7%)	Reference	0.03	Reference	0.04
□ Nonoedematous	183/227 (80.6%)	44/227 (19.4%)	1.63 (1.04, 2.55)		1.64 (1.03,2.64)	
Nutritional status at discharge						
□ No SAM	325/364 (89.3%)	39/364 (10.7%)	Reference	0.41	Reference	0.58
□ SAM	235/285 (82.5%)	50/285 (17.5%)	1.21 (0.77, 1.90)		1.14 (0.72,1.81)	
Discharge HAZ, mean (SD)	−3.0 (1.5)	−3.7 (1.7)	0.81 (0.70, 0.93)	0.002	0.82 (0.71,0.95)	0.007
Chronic underlying conditions						
□ No cerebral palsy	540/619 (87.2%)	79/619 (12.8%)	Reference	0.001	Reference	0.001
□ Cerebral palsy	20/30 (66.7%)	10/30 (33.3%)	3.06 (1.54, 6.08)		2.96 (1.56,5.61)	

*Note*: Data are *n* (row %) unless stated. Univariable analysis was carried out to determine the strength of association of each of the 17 variables; country, residence, sex, age, oedema at hospitalisation, HIV status, duration of hospitalisation, stunting, HAZ, cerebral palsy, haemoglobin, premature cessation of breastfeeding, SAM at discharge, tuberculosis at discharge, caregiver education, caregiver marital status and toilet type with incidence of readmission using a cut‐off of *p *< 0.25. The five variables that were significant at *p* < 0.25 (duration of hospitalisation, HAZ, premature cessation of breastfeeding, toilet type and TB at discharge) and the seven a priori variables (country, sex, age at discharge, HIV status, baseline oedema, SAM at discharge and cerebral palsy) were offered up to the multivariable model. The table shows the adjusted hazards (cox regression model) and adjusted subhazard ratios obtained from the final model where death and loss to follow up were treated as competing risks. Duration of hospitalisation, premature cessation of breastfeeding, TB at discharge and toilet type were not retained in the final model.

Abbreviations: aSHR, adjusted subhazard ratio from competing risks model (treating death and loss to follow up before readmission as competing risks); HAZ, height‐for‐age Z‐score; IQR, Interquartile range; SAM: severe acute malnutrition; SD, standard deviation; TB, tuberculosis.

### 3.5 Morbidity

Nearly half (48.8%) of children had at least one caregiver‐reported symptom of ill‐health within 4 weeks of discharge, with a gradual decline at later follow‐up visits. Fever and respiratory illnesses were the most commonly reported symptoms overall; both declined markedly between 24 and 52 weeks. There were more caregiver‐reported symptoms in HIV‐positive versus HIV‐negative children [63.9% [(95% CI: 53.7%, 73.0%) vs. 44.7% (39.6%, 49.9%); *p* = 0.001] at Week 4, driven particularly by differences in weight loss and appetite, but groups were otherwise similar during follow‐up. There was a significant interaction between HIV status and reported weight loss [OR: 0.71 (0.57,0.90) *p* = 0.004] as well as diarrhoea [OR: 0.83 (0.70, 0.99) *p* = 0.036] over time.

### 3.6 Nutritional recovery

**Figure 2:**
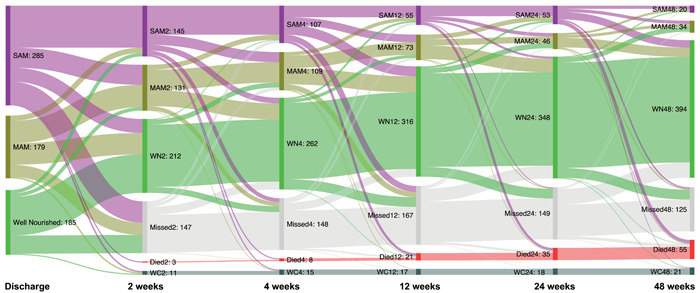
Patterns of nutritional recovery among children discharged from hospital after treatment for complicated severe acute malnutrition (SAM). Sankey diagram showing the pattern of nutritional recovery between hospital discharge and 52 weeks postdischarge. Nutritional status was assessed at every study visit and classified as SAM, moderate acute malnutrition (MAM) or adequately nourished (AN). The purple colour represents SAM, yellow MAM, and green AN. Children who withdrew consent were classified as (WC) and those who missed visits but turned up later were classified as missed

We next looked at the nutritional status over the follow‐up period (Figure 2). At the time of hospital discharge, 43.9% (95% CI: 40.1, 47.8) of children had SAM, 27.6% (24.3, 31.2) had MAM and 28.5% (25.2, 32.1) were well‐nourished. The proportion of children with SAM declined to 22.4% (95% CI: 18.9, 26.4) by Week 4, 12.4% (9.6, 15.8) by Week 12, 11.9% (9.2, 15.2) by Week 24, and 4.5% (2.9, 6.8) by Week 52 Overall, 28.8% (95% CI: 24.8, 33.2) and 12.1% (9.4, 15.5) of children were still undernourished (i.e., WHZ <−2; i.e., SAM or MAM) 24 and 52 weeks after discharge from hospital, respectively. While the proportion with nutritional recovery at 52 weeks was similar between HIV‐positive and HIV‐negative children (88.6% vs. 87.8%, respectively), HIV‐positive children generally had a slower recovery rate following hospital discharge (Figure S2). There was a significant difference in the proportion of children classified as SAM, MAM and adequately nourished between HIV‐positive and HIV‐negative children at Week 2 (*p* = 0.003) and Week 4 (*p* = 0.02), but no significant difference between groups from Week 12 onwards.

**Figure 3:**
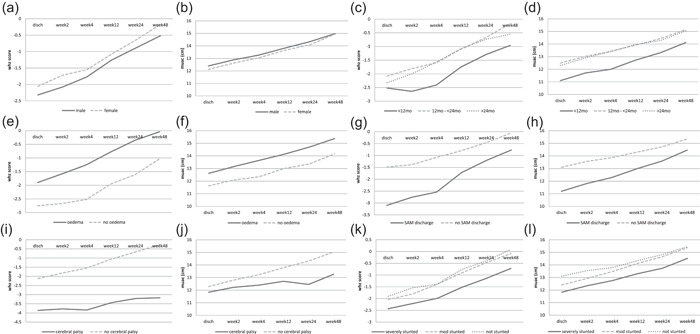
Mean weight for height Z‐score (WHZ) and mid‐upper arm circumference (MUAC) trend over the follow‐up time. Graphs showing mean change in WHZ and MUAC over the period of follow up for the following variables; sex, age, baseline oedema, severe acute malnutrition at discharge, cerebral palsy and stunting

Table S3 shows the regression model of nutritional recovery using WHZ and MUAC as outcome factors and Figure 3 shows the trend in mean WHZ and MUAC over the period of follow‐up. Females compared to males had a better recovery by WHZ, while males had a better recovery when MUAC was used. Young age at discharge (<12 months) was associated with poor recovery throughout the period of follow‐up compared to older children. HIV‐positive compared to HIV‐negative children had a faster recovery in WHZ compared to MUAC. Children who had nonoedematous SAM at hospitalisation recovered at a slower rate than those who had oedema; similarly, children with ongoing SAM at discharge recovered more slowly than those who did not have SAM at discharge. Recovery of children with cerebral palsy remained poor through the period of follow‐up.

We identified six independent factors associated with moving from a lower to a higher nutritional status in the multivariable ordinal logistic regression model (Table S4). Two factors (age at discharge and HIV) were associated with early recovery (at Weeks 2 and 4 only), while four factors (nonoedematous SAM at hospitalisation, ongoing SAM at discharge, cerebral palsy and HAZ) were consistently associated with recovery throughout the follow‐up period. Every 1‐month increase in age was associated with a 4% higher odds (aOR: 1.04, 95% CI: 1.01, 1.06) of moving from a lower to a higher nutritional status at 4 weeks, while HIV was associated with a 41% lower odds (aOR: 0.59, 95% CI: 0.36, 0.97) of moving to a higher nutritional status. Children with nonoedematous compared to oedematous SAM had 71% lower odds (aOR: 0.29, 95% CI: 0.19, 0.44) of moving from a lower to a higher nutritional status at Week 4. Children with ongoing SAM at hospital discharge had 86% lower odds (aOR: 0.14, 95% CI: 0.09, 0.21) and children with cerebral palsy had an 84% lower odds (aOR: 0.16, 95% CI: 0.06, 0.40) of moving to a higher nutritional status by Week 4. Finally, in this cohort where 78% of the children were stunted, every unit increase in HAZ was associated with a 40% increased odds of moving from a lower to a higher nutritional status (aOR: 1.40, 95% CI: 1.20, 1.61) by Week 4. Effect size estimates for the four consistent risk factors remained similar throughout follow‐up, except for the association between SAM at discharge and nutritional recovery, which attenuated over time (Table S4). Sex, being on TB medication, premature cessation of breastfeeding, and household toilet type were not significantly associated with nutritional recovery at any time point.

## 4 DISCUSSION

Children discharged from the hospital after the management of complicated SAM continue to be at high risk of illness, hospital readmission and poor nutritional recovery. In this longitudinal study of children followed up after hospital discharge for SAM at three sites in southern Africa, one‐in‐six were readmitted into hospital over the first year postdischarge. Children had frequent illness episodes, and one‐in‐eight children remained undernourished by 52 weeks postdischarge. We identified four consistent risk factors for hospital readmission and nutritional recovery over 1 year of follow up: nonoedematous SAM at hospitalisation, ongoing SAM at discharge, discharge HAZ and underlying cerebral palsy. Contrary to our initial hypothesis, and despite our previous finding that it is a predictor of postdischarge mortality (Bwakura‐Dangarembizi et al., 2021), HIV was not associated with time to hospital readmission. However, HIV infection and young age at discharge were associated with poor nutritional recovery assessed using WHZ or MUAC. Overall, our data define clear vulnerable population groups among children recovering from complicated SAM and identify risk factors that are congruent with our previous analyses of postdischarge mortality risk in the same cohort (Bwakura‐Dangarembizi et al., 2021). Children with nonoedematous SAM, stunting and disability in particular need to be considered for targeted convalescent care after discharge from hospital following complicated SAM. Infants below 12 months of age should be particularly prioritised for care during the period of postdischarge convalescence, given their slower recovery compared to older children.

WHO guidelines recommend that complicated SAM should be managed in hospital, followed by referral to a community‐based nutritional rehabilitation facility (World Health Organisation, 1999, 2013). Adherence to this model of care depends on functional health systems and access to resources. The period from hospital discharge to nutritional recovery is fraught with vulnerabilities from ongoing undernutrition, unresolved infections, hospital‐acquired infections, perturbed physiological pathways and ongoing immune impairment (Ngari et al., 2018). Children discharged from hospitals in sub‐Saharan Africa following SAM have a particularly high risk of mortality and hospital readmission (Gonzales et al., 2019), with several comorbidities (including HIV) being recognised risk factors in previous studies (Wiens et al., 2013, 2015). Interestingly, HIV infection was not a risk factor for readmission in this cohort, though it was one of the major risk factors we previously identified for postdischarge mortality (Bwakura‐Dangarembizi et al., 2021). It is possible that the higher death rate among children with HIV, almost half of which occurred in the community, proportionately reduced the number of readmissions in this group. Alternatively, HIV‐positive children may be protected from readmission (but not mortality) by cotrimoxazole prophylaxis, since hospitalisation in children with malnutrition is usually triggered by infections (Chintu et al., 2004; Mulenga et al., 2007). By contrast, cotrimoxazole prophylaxis did not reduce mortality or hospital readmissions in HIV‐negative children discharged from hospital following complicated SAM in Kenya (Berkley et al., 2016). Low HAZ is an indicator of poor linear growth and was an independent risk factor for hospital readmission. Diagnosis of SAM does not take into account stunting (HAZ <−2), which reflects chronic malnutrition associated with multiple causes such as poverty, food insecurity, repeated infections and intrauterine growth restriction (Stobaugh, Rogers, et al., 2018). There is increasing evidence that children with concurrent wasting and stunting are at greater risk for both mortality and morbidity (McDonald et al., 2013; Stobaugh, Rogers, et al., 2018; Wells et al., 2019). This increased risk is hypothesised to be mediated through decreased muscle mass and fat mass (seen in both wasting and stunting) resulting in low levels of the hormone leptin, which has a key role in both innate and adaptive immunity (Briend et al., 2015). Our observation that HAZ is associated with hospital readmission supports the need to better understand the pathophysiology of stunting in children with SAM. The observation that children enroled in Zambia had a longer time to first hospital readmission compared to children enroled in Zimbabwe, may be a result of continued nutritional support once children were categorised with MAM, which was not the practice in Zimbabwe.

This study reports the pattern of illness during follow‐up after complicated SAM. Consistent with other studies, fever, respiratory illness and diarrhoea were the most commonly reported symptoms postdischarge (Ashraf et al., 2012; Khanum et al., 1998). The pattern of morbidity in children treated for SAM, therefore, reflects the typical infectious disease burden seen in LMICs. Our cohort showed a sharp decline in reported symptoms after 24 weeks of follow‐up, suggesting that the first 6 months following discharge is a critical time for comorbidities. The high prevalence of illness during these first few months highlights the need for continued surveillance and interventions to prevent and treat infections after discharge. It has long been proposed that a cycle exists whereby malnutrition impairs immunity, thereby increasing host susceptibility to infection, which in turn intensifies the severity of malnutrition (Katona & Katona‐Apte, 2008; Kau et al., 2011; Scrimshaw et al., 1968; Walson & A, 2018). This is a hypothesis that is being explored using biological specimens collected from HOPE‐SAM participants (Bwakura‐Dangarembizi et al., 2019). The effect of antimicrobials in children recovering from complicated SAM requires further study, especially since they may have additional benefits for weight gain and nutritional recovery (Trehan et al., 2013). For example, an ongoing study among children with uncomplicated SAM in Burkina Faso is providing azithromycin as an adjunct to standard outpatient treatment (ClinicalTrials. gov Identifier: NCT03568643).

At the time of hospital discharge, 44% of children had ongoing SAM. Most studies on nutritional recovery evaluate children after discharge from community‐based therapeutic feeding centres, thus missing the immediate postdischarge period (Khanum et al., 1998; Somassè et al., 2016). Even then, there is no standard definition of relapse following treatment of SAM from existing studies due to different follow‐up periods, treatment protocols and inconsistent reporting of point prevalence, cumulative prevalence and incidence rates (Stobaugh, Mayberry, et al., 2018). Our study showed that 6 months after discharge from hospital, 22% of children had not achieved nutritional recovery (here defined as improvement in anthropometry from SAM to either MAM or adequately‐nourished, or from MAM to adequately‐nourished). One‐year after discharge, 12% of children still had MAM or SAM. Consistent with our observation that ongoing SAM at discharge was a predictor of poor nutritional recovery, a study of Ethiopian children with MAM who did not receive supplementary feeds, showed that the most wasted children at baseline had the lowest chance of recovering after 28 weeks of follow‐up (James et al., 2016). Previous studies have found a strong association between poor linear growth and nutritional recovery in children with MAM and SAM (Chang et al., 2013; Stobaugh, Rogers, et al., 2018). Management of SAM is the same regardless of whether or not children are stunted. However, children who have both SAM and stunting may need additional interventions as the overlapping effects of stunting and wasting compound the risk of poor outcomes (Isanaka et al., 2019; McDonald et al., 2013; Stobaugh, Rogers, et al., 2018).

Our study showed that HIV infection slowed nutritional recovery in the early weeks following hospital discharge; at Weeks 2 and 4 postdischarge children with HIV had a lower odds of nutritional recovery, but this effect was not seen from Week 12 onwards. Overall our findings are similar to a study by Fergusson et al. (2009) from Malawi, where nutritional recovery of HIV‐positive and HIV‐negative children was similar at 4 months after discharge from hospital. Unlike the children in our cohort (in whom 28.5% had a WHZ > −2 at discharge), children in the Malawi cohort were discharged after attaining a WHZ of 85% (equivalent to WHZ ≥ −1). The odds of nutritional recovery were extremely poor in children with cerebral palsy. Children with disabilities are at risk for poor nutritional status, especially if they have a severe gross motor impairment and oropharyngeal dysfunction, leading to reduced food intake, increased nutrient loss, additional nutrient requirements and significantly increased energy expenditure (Bell & Samson‐Fang, 2013). In the analysis models where MUAC and WHZ were used as continuous variables, young age at discharge, nonoedematous SAM at the time of initial hospitalisation, SAM at discharge and cerebral palsy were associated with poor nutritional recovery throughout the period of follow‐up. This indicates that the public health approach which uses cut‐offs for nutritional status may miss children who have poor nutritional recovery. HIV‐positive children had slower recovery than HIV‐negative children up to 4 weeks postdischarge. Taken together, our study provides clear evidence that a proportion of children do not achieve nutritional recovery 1 year after discharge from hospital, with severe wasting, disability and stunting as key risk factors. Our findings support what Stobaugh described as 'different trajectories of recovery', suggesting that uniform outpatient management for all children recovering from SAM may not ensure sustainable recovery (Stobaugh, Mayberry, et al., 2018). Here we demonstrate that existing nutritional rehabilitation is insufficient to promote full and lasting recovery in all children. Future studies need to evaluate whether enhanced convalescent care and/or adjunctive therapies improve outcomes for the children most vulnerable to relapse and readmission.

Our study has strengths and limitations. We achieved relatively high rates of follow‐up among this highly mobile and vulnerable population compared to other studies, through phone calls, text messages, free study clinics and home visits. However, we were not able to collect anthropometry and morbidity data on 15.8% of the study population at 52 weeks, which could have biased our findings. Where there were missing risk factors for analysis, imputation led to no major changes in the inferences obtained. We relied on caregiver‐reported data for children who were hospitalised at nonstudy sites, which may have underestimated the proportion of children readmitted. A major limitation was that we did not systematically record nutritional rehabilitation provided in the community. We collected data on the home environment but we may not have captured key factors associated with clinical outcomes such as feeding practices, household food security and caregiver health‐seeking behaviours. An in‐depth social science study of the same population is underway to address this issue. Relapse of malnutrition may have been underestimated as we were only able to capture events at the time of the study visits. For example, it is well known that nutritional oedema can be transient and mild cases could have resolved between study visits (Alvarez et al., 2016).

Few longitudinal studies have defined morbidity and nutritional recovery of children discharged from hospital for complicated SAM. Here, we found high rates of hospital readmission and caregiver‐reported illness in the first 6 months after discharge, and failure to achieve nutritional recovery by 1‐year postdischarge in 12% of the cohort. The current study, together with our previous findings on mortality in the same cohort, identify a small number of consistent risk factors for adverse outcomes following discharge from hospital: HIV, nonoedematous SAM, cerebral palsy and ongoing SAM at the time of discharge (Bwakura‐Dangarembizi et al., 2021). The current classification of malnutrition into acute and chronic and subsequent approaches to management ignores the contribution of poor linear growth to the poor outcomes in children who are both wasted and stunted namely relapse of malnutrition and associated morbidity (Stobaugh, Rogers, et al., 2018). HAZ should be considered in identifying severely malnourished children who are at risk for poorer outcomes. Prioritising these high‐risk groups for more intensive follow‐up may be beneficial. Providing additional and holistic convalescent care for all children discharged from hospitals following SAM may contribute to improving long‐term outcomes for this vulnerable population.

## CONFLICT OF INTERESTS

The authors declare that there are no conflict of interests.

## AUTHOR CONTRIBUTIONS

Mutsa Bwakura‐Dangarembizi, Beatrice Amadi, Kusum J. Nathoo, Shane A. Norris, Paul Kelly and Andrew J. Prendergast designed and oversaw the research study. Mutsa Bwakura‐Dangarembizi, Beatrice Amadi, Claire D. Bourke, Ruairi C. Robertson, Kusum J. Nathoo, Paul Kelly and Andrew J. Prendergast secured funding for the study. Mutsa Bwakura‐Dangarembizi, Kanta Chandwe, Chanda Kapoma, Beatrice Amadi, Deophine Ngosa and Florence D. Majo collected the data. Mutsa Bwakura‐Dangarembizi, Andrew J. Prendergast, Cherlynn Dumbura, Bernard Chasekwa, Jonathan P. Sturgeon, Claire D. Bourke, Ruairi C. Robertson and Robert Ntozini cleaned, analysed and interpreted the data. Mutsa Bwakura‐Dangarembizi and Andrew J. Prendergast wrote the first draft of the paper. All authors critically revised the manuscript.

## Supporting information

Supporting information.

Supporting information.

Supporting information.

Supporting information.

## Data Availability

The data that support the findings of this study are available from the corresponding author upon request.
