# Supplementary material for: Recovery of children following hospitalisation for complicated severe acute malnutrition
Source: Matern Child Nutr. 2021 Dec 22;18(2):e13302. doi: 10.1111/mcn.13302 (PMC8932709; doi:10.1111/mcn.13302)
Supplement: Supplementary file 1 — Supporting information. [file MCN-18-e13302-s003.docx]

**SUPPLEMENTARY METHODS**

**Recovery of children following hospitalization for complicated severe acute malnutrition in Zimbabwe and Zambia**

Mutsa Bwakura-Dangarembizi^1,2^, Cherlynn Dumbura^2^, Beatrice Amadi^3^, Bernard Chasekwa^2^, Deophine Ngosa^3^, Florence D. Majo^2^, Jonathan P. Sturgeon^2,4^, Kanta Chandwe^3^, Chanda Kapoma^3^, Claire D. Bourke^2,4^, Ruairi C. Robertson^2,4^, Kusum J. Nathoo^1^, Robert Ntozini^2^, Shane A Norris^5^, Paul Kelly^3,4^, Andrew J. Prendergast^2,4^ on behalf of the HOPE-SAM study team*

^1^ Department of Paediatrics and Child Health, University of Zimbabwe College of Health Sciences, Harare, Zimbabwe

^2^Zvitambo Institute for Maternal and Child Health Research, Harare, Zimbabwe

^3^Tropical Gastroenterology and Nutrition Group, University of Zambia, Lusaka, Zambia

^4^Blizard Institute, Queen Mary University of London, London, UK

^5^University of the Witwatersrand, Johannesburg, South Africa

1. **Selection of exposure variables**

Selection of exposure variables for time to first hospitalization was based on biological plausibility and included the following; country, residence, sex, age, edema at hospitalization, HIV status, duration of hospitalization, stunting, HAZ, cerebral palsy, haemoglobin, premature cessation of breastfeeding, SAM at discharge, tuberculosis at discharge, caregiver education, caregiver marital status and toilet type. These were evaluated in univariable analysis for strength of association and retained if P <0.25. Twelve variables (tuberculosis at discharge, premature cessation of breastfeeding [defined as stopping breastfeeding before 12 months of age], duration of hospitalization, HAZ at discharge, toilet type and 7 a-priori variables (age, country, sex, cerebral palsy, SAM at discharge, HIV status and edema at hospitalization) were included in the final model.

**Supplementary Table 1: Admission characteristics of children who did not attend any visit and those who attended at least one visit**

|  | **Last seen at discharge**  **N=71** | **Attended at least one visit**  **N=578** | **P-value^a^** |
| --- | --- | --- | --- |
|  |  |  |  |
| **Country**   - **Zambia** - **Zimbabwe** | 19/71 (26.8%)  52/71 (73.2%) | 169/578 (29.2%)  409/578 (70.8%) | 0.66 |
| **Male** | 31/71 (43.7%) | 313/578 (54.2%) | 0.10 |
| **Age, months; median (IQR)** | 18.6 (14.4, 22) | 18.0 (13.5, 22.7) | 0.94 |
| **HIV status**   - **Positive** | 10/71 (14.1%) | 120/578 (20.8%) | 0.19 |
| **Previous history**   - **Complicated SAM** - **Uncomplicated SAM** - **Persistent diarrhea past 2 weeks** | 10/70 (14.3%)  13/69 (18.8%)  15/62 (24.2%) | 82/568 (14.4%)  111/589 (20.1%)  108/528 (20.5%) | 0.97  0.81  0.49 |
| **Currently breastfeeding**  **Premature breastfeeding cessation <12mo**   - **Duration of breastfeeding mo; median (IQR)** | 14/71 (19.7%)  13/57 (22.3%)  9 (9, 9) | 114/572 (19.9%)  149/458 (32.5%)  8 (5, 9) | 0.97  0.14  0.10 |
| **Type of SAM at admission**   - Oedematous | 52/71 (73.2%) | 370/578 (64.0%) | 0.12 |
| **Nutritional status at discharge**   - SAM at discharge | 29/71 (40.9%) | 256/578 (44.3%) | 0.58 |
| **Anthropometry**  WHZ, mean (SD)  WAZ, mean (SD)  HAZ, mean (SD)  MUAC, cm; mean (SD) | -1.95 (1.3)  -3.10 (1.5)  -3.01 (1.4)  12.5 (1.8) | -2.23 (1.5)  -3.36 (1.6)  -3.11 (1.6)  12.2 (1.6) | 0.13  0.19  0.60  0.26 |
| **Stunting at discharge**   - Yes | 55/71(77.5%) | 449/578 (77.7%) | 0.97 |
| **Duration of hospitalisation, days; median (IQR)** | 6 (4, 10) | 7 (4, 13) | 0.14 |
| **Chronic underlying conditions**   - Cerebral palsy - Hydrocephalus - Congenital heart disease   **Medications at discharge**   - TB medication - ART medication(HIV positive only) | 1/71 (1.4%)  0/71 (0.0%)  1/71 (1.4%)  4/71 (5.6%)  5/10 (50.0%) | 29/578 (5.0%)  3/578 (0.5%)  13/578 (2.3%)  84/578 (14.5%)  61/120 (50.8%) | 0.17  0.54  0.65  0.04  0.96 |
| **Haemoglobin, g/dL; median (IQR)^b^** | 9.2 (8.4, 10.6) | 9.3 (8.2, 10.3) | 0.56 |
| **Primary caregiver**   - Mother   **Age,** years; median (IQR)    **Marital status**   - Married/stable union   **Education**, years; median (IQR)  **Employment**   - None - skilled - unskilled   **Residence**   - Rural - Urban - Peri-urban   **Drinking water source**   - Improved   **Toilet facilities**   - Improved - Unimproved - None   **Household electricity**   - Yes | 68/69 (98.6%)  23 (21, 28)  51/69 (73.9%)  9 (7, 11)  37/69 (53.6%)  7/69 (10.1%)  25/69 (36.2%)  9/70 (12.9%)  43/70 (61.4%)  18/70 (25.7%)  66/70 (94.3%)  62/69 (89.9%)  5/69 (7.3%)  2/69 (2.9%)  33/66 (50.0%) | 522/564 (92.6%)  27 (23, 31)  420/564 (74.5%)  10 (8, 11)  336/562 (59.8%)  39/562 (6.9%)  186/562 (33.1%)  90/575 (15.7%)  361/575 (62.8%)  124/575 (21.6%)  533/571 (93.4%)  506/574 (88.2%)  46/574 (8.0%)  22/574 (3.8%)  262/568 (46.1%) | 0.06  <0.001  0.92  0.66  0.49  0.66  0.76  0.90  0.55 |

Data are n (column %) unless stated. ^a^ p value comparing those who never attended a visit post discharge and those who attended one or more visits.

Chi squared test was used to compare categorical variables and t test was used to compare continuous variables.

HAZ: height-for-age Z-score; IQR: Interquartile range, SAM: severe acute malnutrition, SD: standard deviation; TB: tuberculosis

**Supplementary Table 2: Incident rate ratios for hospital readmission over 52 weeks of follow-up**

|  | **N** | **Readmission rate per 100 child-weeks (95%CI)** | **Unadjusted IRR (95%CI)** | **P value** | **Adjusted IRR (95%CI)** | **P-value** |
| --- | --- | --- | --- | --- | --- | --- |
| **Country**   - Zimbabwe - Zambia | 461  188 | 0.45 (0.35, 0.57)  0.33 (0.21, 0.51) | Reference  0.75 (0.45, 1.25) | 0.28 | Reference  0.62 (0.37, 1.05) | 0.08 |
| **Sex**   - Male - Female | 344  305 | 0.48 (0.37, 0.63)  0.34 (0.24, 0.48) | Reference  0.69 (0.44, 1.07) | 0.10 | Reference  0.69 (0.44, 1.08) | 0.11 |
| **Age at discharge (mo), median (IQR)** | - | - | 0.98 (0.95, 1.01) | 0.12 | 0.98 (0.95, 1.01) | 0.12 |
| **HIV status**   - HIV-positive - HIV-negative | 130  519 | 0.53 (0.35, 0.80)  0.39 (0.30, 0.49) | 1.17 (0.69, 2.00)  Reference | 0.56 | 1.22 (0.71, 2.11)  Reference | 0.47 |
| **Type of SAM at hospitalization**   - Non-edematous - Edematous | 227  422 | 0.63 (0.47, 0.83)  0.31 (0.23, 0.42) | 2.09 (1.35, 3.23)  Reference | 0.001 | 1.65 (1.05, 2.61)  Reference | 0.03 |
| **Discharge Anthropometry**   - HAZ | - | - | 0.75 (0.65, 0.86) | <0.001 | 0.81 (0.71, 0.94) | 0.005 |
| **Nutritional status at discharge**   - SAM - No SAM | 285  364 | 0.57 (0.43, 0.75)  0.31 (0.23, 0.42) | 1.96 (1.26. 3.04)  Reference | 0.003 | 1.33 (0.84, 2.11)  Reference | 0.23 |
| **Cerebral palsy**   - Yes - No | 30  619 | 1.33 (0.72, 2.47)  0.38 (0.31, 0.48) | 2.31 (1.00, 5.35)  Reference | 0.05 | 2.02 (0.89, 4.59)  Reference | 0.09 |

Univariable and multivariable negative binomial models were used to calculate the incidence rate ratio (IRR) for readmission through 52 weeks. Univariable analysis was carried out to determine strength of association of each of the seventeen variables; country, residence, sex, age, edema at hospitalization, HIV status, duration of hospitalization, stunting, HAZ, cerebral palsy, haemoglobin, premature cessation of breastfeeding, SAM at discharge, tuberculosis at discharge, caregiver education, caregiver marital status and toilet type with incidence of readmission using a cut-off of P < 0.25.

The three variables that were significant at P <0.25 (duration of hospitalization, HAZ, and TB at discharge) and 7 variables selected for inclusion *a priori* on the basis of their known association with post-discharge mortality (country, sex, age at discharge, HIV status, baseline edema, SAM at discharge and cerebral palsy) were offered up to the final multivariable model. The table shows the 8 variables that were retained in the final multivariable model. TB at discharge and duration of hospitalization were not retained in the final model

|  | **Week 2** | | **Week 4** | | **Week 12** | | **Week 24** | | **Week 48- 52** | |
| --- | --- | --- | --- | --- | --- | --- | --- | --- | --- | --- |
|  | **WHZ** | **MUAC** | **WHZ** | **MUAC** | **WHZ** | **MUAC** | **WHZ** | **MUAC** | **WHZ** | **MUAC** |
|  | **Coef (95% CI)** | **Coef (95% CI)** | **Coef (95% CI)** | **Coef (95% CI)** | **Coef (95% CI)** | **Coef (95% CI)** | **Coef (95% CI)** | **Coef (95% CI)** | **Coef (95% CI)** | **Coef (95% CI)** |
| **Country**: Zambia | 0.34(0.08,0.60) | 0.18(-0.06,0.42) | 0.21(-0.06,0.47) | -0.01(-0.27,0.26) | -0.07(-0.35,0.20) | 0.26(0.54,0.02) | 0.19(-0.09,0.47) | -0.10(-0.39,0.20) | 0.15(-0.15,0.44) | -0.52(-0.80,-0.23) |
| Female | 0.32(0.09,0.54) | -0.30(-0.52,-0.09) | 0.24(0.01,0.46) | -0.26(-0.49,-0.04) | 0.15(-0.08,0.38) | -0.26(-0.49,-0.03) | 0.23 (-0.01,0.47) | -0.31(-0.56,0.05) | 0.20(-0.03,0.44) | -0.16(-0.41,0.08) |
| Age at discharge (mo)  *Age categories  <12mo  12mo-<24mo  >24mo | 0.02(0.01,0.03)  Ref  0.72(0.31,1.14)  0.84(0.37,1.32) | 0.03(0.02,0.04)  Ref  0.67(0.36,0.99)  0.86(0.48,1.23) | 0.02(0.01,0.04)  Ref  0.50(0.12,0.89)  0.83(0.38,1.27) | 0.04(0.03,0.05)  Ref  0.85(0.51,1.18)  1.18(0,78,1.59) | 0.02(0.00,0.03)  Ref  0.40(0.03,0..76)  0.62(0.19,1.05) | 0.04(0.02,0.05)  Ref  0.75(0.40,1.10)  1.10(0.68,1.52) | 0.01(-0.00,0.03)  Ref  0.30(-0.07,0.66)  0.52(0.08,0.96) | 0.03(0.01,0.04)  Ref  0.75(0.37,1.13)  0.98(0.52,1.44) | 0.00(-0.02,0.01)  Ref  0.48(0.13,0.83)  0.30(-0.14,0.74) | 0.02(0.00,0.04)  Ref  0.72(0.35,1.09)  0.86(0.39.1.32) |
| HIV positive | -0.11(-0.41,0.18) | -0.33(-0.61,-0.04) | -0.19 (-0.49,0.11) | -0.37(-0.68,-0.07) | 0.10(-0.21,0.40) | -0.36(-0.66,-0.60) | 0.07(-0.25,0.39) | -0.29(-0.63,-0.05) | 0.59(0.28,0.91) | 0.19(-0.14,0.52) |
| No edema at hospitalization | -0.82 (-1.07,0.58) | -0.48(-0.71,-0.25) | -0.92 (-1.1,-0.68) | -0.67(-0.91,-0.42) | -0.92 -1.17,-0.67) | -0.65(-0.90,-0.40) | -1.08(-1.34,-0.82) | -0.96(-1.22,-0.67) | -0.90(-1.16,-0.64) | -0.89(-1.16,-0.62) |
| SAM at discharge | -1.13 (-1.37,0.90) | -1.28-1.51,-1.06) | -1.17(-1.40,-0.94) | -1.10(-1.33,-0.87) | -0.67 (-0.91-0.43) | -0.84(-1.08,-0.60) | -0.46(-0.71,-0.21) | 0.64(-0.91,-0.38) | -0.42(-0.66,-0.17) | -0.51(-0.77,-0.26) |
| Discharge HAZ  *HAZ categories  HAZ>=-2  HAZ -3 to <-2  HAZ <-3 | 0.15 (0.06,0.23)  Ref  -0.43(-0.75,-0.12)  -0.49(-0.79,-0.19) | 0.33(0.25,0.40)  Ref  -0.78(-1.08,-0.46)  -0.96(-1.24,-0.68) | 0.13(0.04,0.21)  Ref  -0.17(-0.48,0.14)  -0.34(-0.62,-0.04) | 0.30(0.22,0.37)  Ref  -0.50(-0.93,-0.17)  -0.80(-1.11,-0.51) | 0.17(0.08,0.26)  Ref  -0.28(-0.60,0.05)  -0.49(-0.79,-0.19) | 0.32(0.24,-.40)  Ref  -0.36(-0.70,-0.02)  -0.80(-1.11,-0.49) | 0.18(0.10,0.27)  Ref  -0.16(-0.50,0.18)  -0.51(-0.82,-0.19) | 0.35(0.26,0.43)  Ref  -0.30(-0.67,0.07)  -0.89(-1.23,-0.55) | 0.20(0.11,0.28)  Ref  -0.27(-0.60,0.06)  -0.57(-0.87,-0.26) | 0.29(0.21,0.38)  Ref  -0.22(-0.57,0.13)  -0.79(-1.11,-0.47) |
| Cerebral palsy | -1.58 (-2.08,1.08) | -0.25(-0.73,0.23) | -1.84(-2.34,-1.34) | -0.69(-1.22,-0.15) | -1.95(-2.48,-1.41) | -0.69(-1.22,-0.15) | -2.20(2.80,-1.60) | -1.46(-2.08,-0.84) | -2.35(-2.99,-1.71) | -1.34(-2.01,-0.67) |

**Supplementary Table 3. Regression model of nutritional recovery using WHZ and MUAC**

Coefficients of nutritional recovery were estimated using a regression model with WHZ and MUAC as continuous outcomes.

Univariable analysis was carried out to determine strength of association of each of the seventeen variables; country, residence, sex, age, edema at hospitalization, HIV status, duration of hospitalization, stunting, HAZ, cerebral palsy, haemoglobin, premature cessation of breastfeeding, SAM at discharge, tuberculosis at discharge, caregiver education, caregiver marital status and toilet type with incidence of readmission using a cut-off of P < 0.25.

The 5 variables that were significant at p <0.25 (duration of hospitalization, HAZ, premature cessation of breastfeeding, toilet type and TB at discharge) and the 7 a priori variables (country, sex, age at discharge, HIV status, baseline edema, SAM at discharge and cerebral palsy) were offered up to the regression model.

The table shows the adjusted coefficients obtained from the final model at each time-point.

SAM: severe acute malnutrition, HAZ: height-for-age Z-score; TB: tuberculosis; WHZ: weight-for-height Z-score, MUAC: mid-upper-arm circumference

**Supplementary Table 4. Ordinal Logistic regression model of nutritional recovery**

|  | **Week 2** | | **Week 4** | | | **Week 12** | | | **Week 24** | | | **Week 52** | | |
| --- | --- | --- | --- | --- | --- | --- | --- | --- | --- | --- | --- | --- | --- | --- |
|  | **Odds of nutritional**  **recovery**  **Adjusted OR (95%CI)** | **P value** | **Odds of nutritional**  **recovery**  **Adjusted OR (95%CI)** | **P value** | **Odds of nutritional**  **recovery**  **Adjusted OR (95%CI)** | | **P value** | **Odds of nutritional**  **recovery**  **Adjusted OR (95%CI)** | | **P value** | **Odds of nutritional**  **recovery**  **Adjusted OR (95%CI)** | | **P value** |  |
| **Country**   - Zimbabwe - Zambia | Reference  1.50 (1.00, 2.25) | 0.05 | Reference  1.18 (0.76, 1.82) | 0.47 | Reference  0.86 (0.50, 1.46) | | 0.56 | Reference  1.87 (1.04, 3.36) | | 0.04 | Reference  1.29 (0.60, 2.77) | | 0.52 |  |
| **Sex**   - Male - Female | Reference  1.11 (0.76, 1.64) | 0.57 | Reference  1.05 (0.70, 1.57) | 0.83 | Reference  1.09 (0.68, 1.75) | | 0.73 | Reference  1.12 (0.68, 1.84) | | 0.65 | Reference  1.99 (1.00, 3.40) | | 0.05 |  |
| **Age at discharge** | 1.03 (1.01, 1.05) | 0.01 | 1.04 (1.01, 1.06) | 0.003 | 1.01 (0.99, 1.03) | | 0.34 | 1.00 (0.98, 1.04) | | 0.55 | 1.00 (0.97, 1.04) | | 0.84 |  |
| **HIV status**   - HIV-negative - HIV-positive | Reference  0.60 (0.37, 0.96) | 0.03 | Reference  0.59 (0.36, 0.97) | 0.04 | Reference  0.76 (0.43, 1.35) | | 0.36 | Reference  1.04 (0.56, 1.94) | | 0.89 | Reference  1.33 (0.57, 3.10) | | 0.51 |  |
| **Type of SAM at hospitalization**   - edematous - Non-edematous | Reference  0.36 (0.24, 0.54) | <0.001 | Reference  0.29 (0.19, 0.44) | <0.001 | Reference  0.27 (0.16, 0.43) | | <0.001 | Reference  0.31 (0.18, 0.51) | | <0.001 | Reference  0.20 (0.10, 0.41) | | <0.001 |  |
| **Nutritional status at discharge**   - No SAM - SAM | Reference  0.13 (0.09, 0.20) | <0.001 | Reference  0.14 (0.09, 0.21) | <0.001 | Reference  0.21 (0.13, 0.35) | | <0.001 | Reference  0.42 (0.25, 0.70) | | 0.001 | Reference  0.34 (0.17, 0.70) | | 0.003 |  |
| **Discharge HAZ** | 1.47 (1.28, 1.70) | <0.001 | 1.40 (1.21, 1.61) | <0.001 | 1.50 (1.26, 1.79) | | <0.001 | 1.26 (1.07, 1.49) | | 0.006 | 1.56 (1.23, 2.00) | | <0.001 |  |
| **Chronic underlying conditions**   - No cerebral palsy - Cerebral palsy | Reference  0.17 (0.07, 0.43) | <0.001 | Reference  0.16 (0.06, 0.40) | <0.001 | Reference  0.08 (0.03, 0.22) | | <0.001 | Reference  0.10 (0.04, 0.27) | | <0.001 | Reference  0.02 (0.01, 0.06) | | <0.001 |  |

Adjusted odds ratios of nutritional recovery were estimated using an ordinal logistic regression model

Univariable analysis was carried out to determine strength of association of each of the seventeen variables; country, residence, sex, age, edema at hospitalization, HIV status, duration of hospitalization, stunting, HAZ, cerebral palsy, haemoglobin, premature cessation of breastfeeding, SAM at discharge, tuberculosis at discharge, caregiver education, caregiver marital status and toilet type with incidence of readmission using a cut-off of P < 0.25.

The 5 variables that were significant at p <0.25 (duration of hospitalization, HAZ, premature cessation of breastfeeding, toilet type and TB at discharge) and the 7 a priori variables (country, sex, age at discharge, HIV status, baseline edema, SAM at discharge and cerebral palsy) were offered up to the mfp model.

The table shows the adjusted hazard ratios obtained from the final model at each time-point. Duration of hospitalization, premature cessation of breastfeeding, TB at discharge and toilet type were not retained in the final model at any time-point.

SAM: severe acute malnutrition, HAZ: height-for-age Z-score; TB: tuberculosis
