# Supplementary figures and images for: Recovery of children following hospitalisation for complicated severe acute malnutrition
Source: Matern Child Nutr. 2021 Dec 22;18(2):e13302. doi: 10.1111/mcn.13302 (PMC8932709; doi:10.1111/mcn.13302)

Supplementary figure 1: Flow diagram

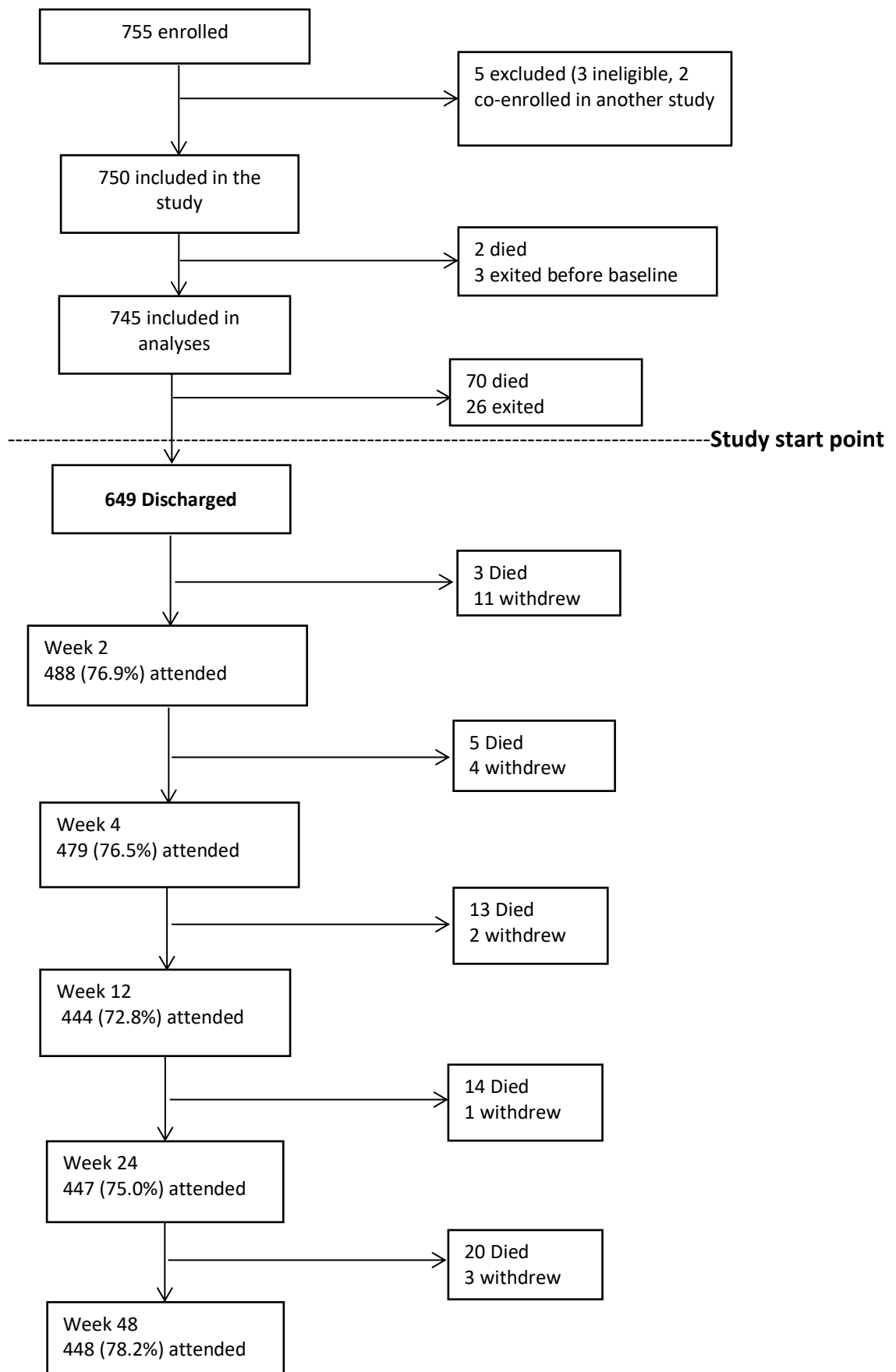

Supplement: Supplementary file 2 — Supporting information. [file MCN-18-e13302-s004.pdf]

# HIV Positive

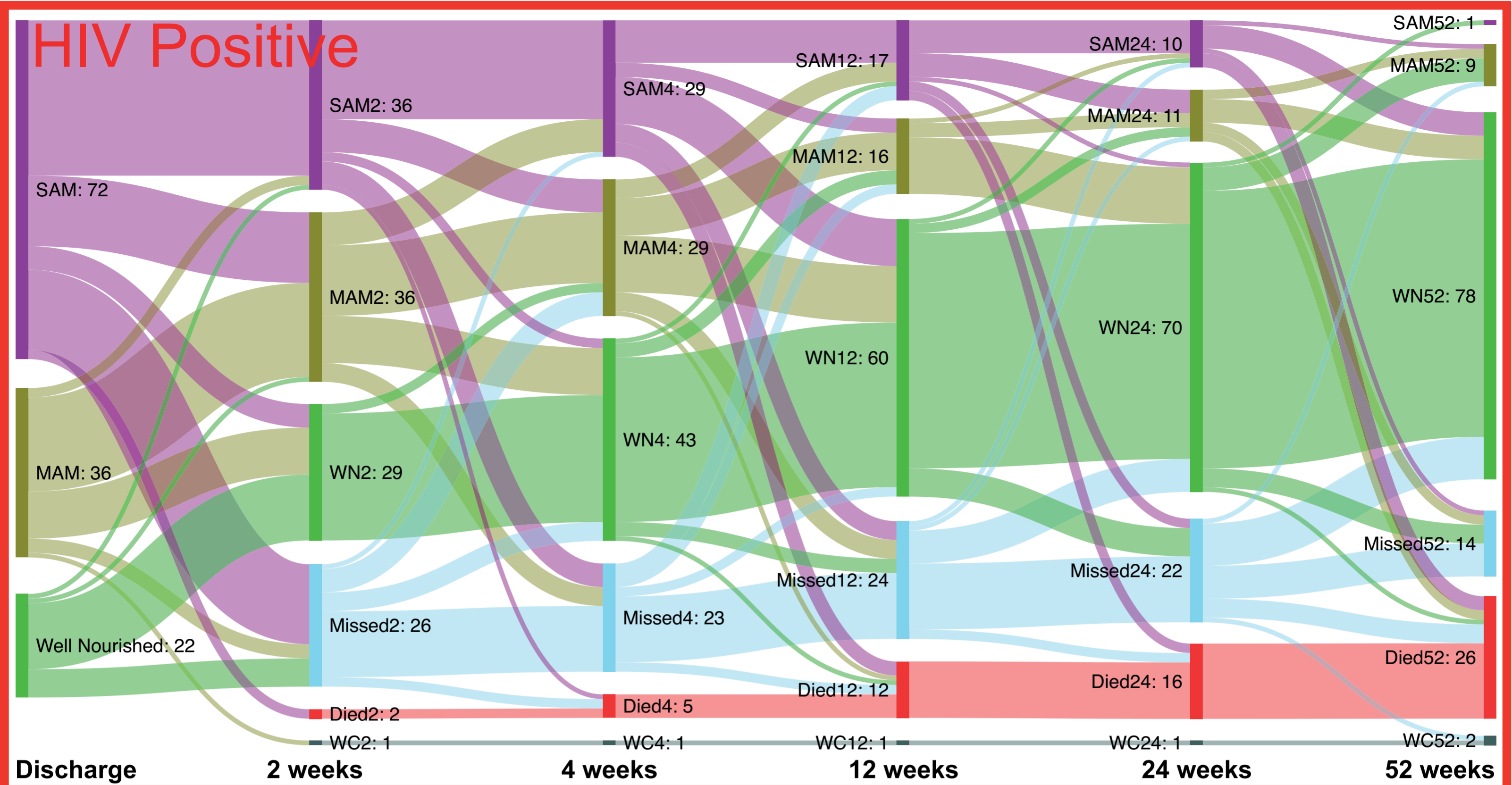

# HIV Negative

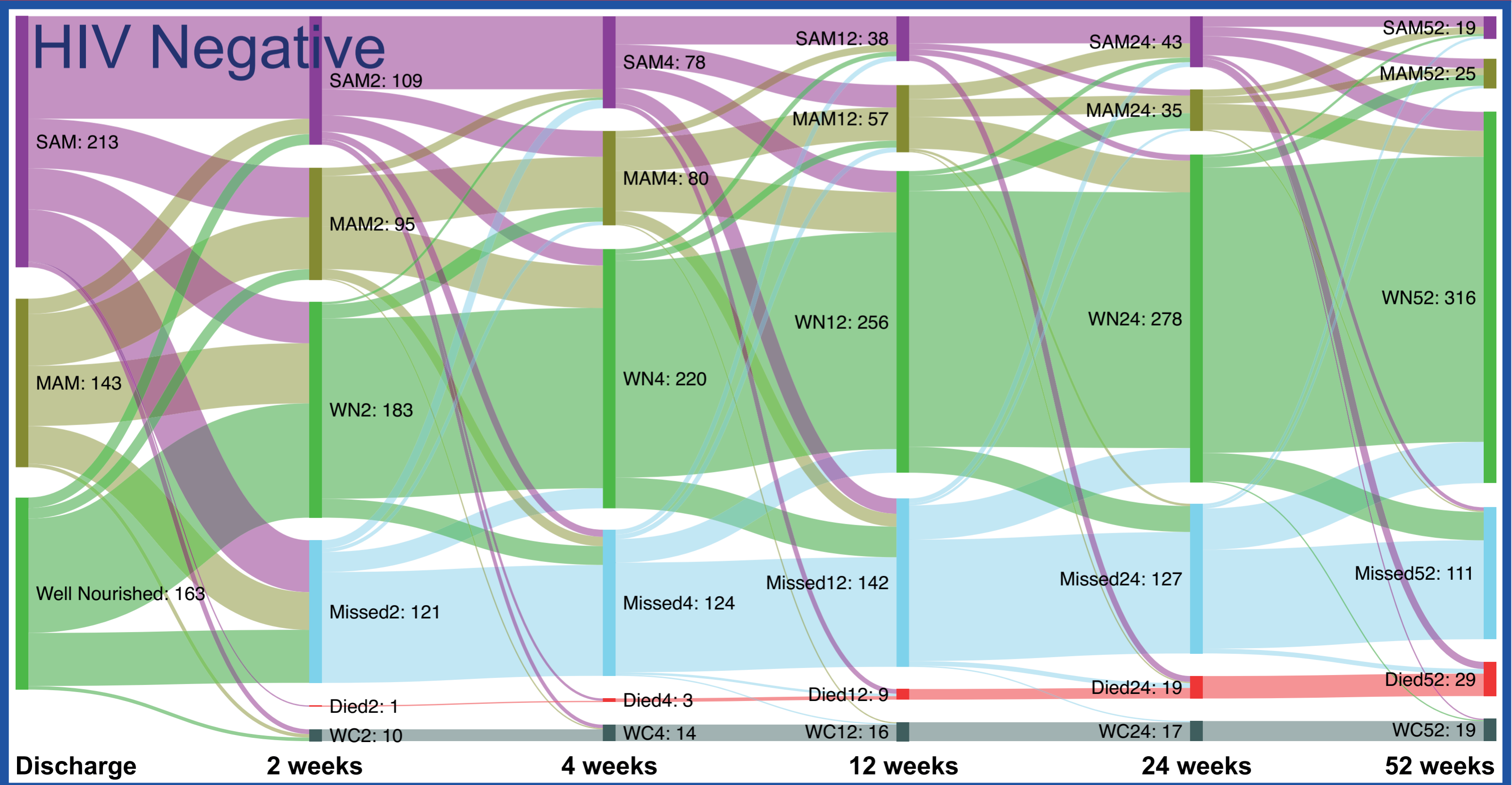

Supplement: Supplementary file 3 — Supporting information. [file MCN-18-e13302-s002.pdf]
