# Supplementary material for: Recovery of children following hospitalisation for complicated severe acute malnutrition
Source: Matern Child Nutr. 2021 Dec 22;18(2):e13302. doi: 10.1111/mcn.13302 (PMC8932709; doi:10.1111/mcn.13302)
Supplement: Supplementary file 4 — Supporting information. [file MCN-18-e13302-s001.docx]

**Supplementary Figure 1**

Flow of participants through the study

**Supplementary Figure 2**

Patterns of nutritional recovery among children discharged from hospital after treatment for complicated SAM stratified by HIV status.
